# Modulation of Toll-Like Receptor Activity by Leukocyte Ig-Like Receptors and Their Effects during Bacterial Infection

**DOI:** 10.1155/2010/536478

**Published:** 2010-06-20

**Authors:** Louise E. Pilsbury, Rachel L. Allen, Martin Vordermeier

**Affiliations:** ^1^Centre for Infection, St George's University of London, Cranmer Terrace, London SW17 0RE, UK; ^2^Veterinary Laboratories Agency, Weybridge, New Haw, KT15 3NB, UK

## Abstract

Toll-like receptors (TLRs) are a potent trigger for inflammatory immune responses. Without tight regulation their activation could lead to pathology, so it is imperative to extend our understanding of the regulatory mechanisms that govern TLR expression and function. One family of immunoregulatory proteins which can provide a balancing effect on TLR activity are the Leukocyte Ig-like receptors (LILRs), which act as innate immune receptors for self-proteins. Here we describe the LILR family, their inhibitory effect on TLR activity in cells of the monocytic lineage, their signalling pathway, and their antimicrobial effects during bacterial infection. Agents have already been identified which enhances or inhibits LILR activity raising the future possibility that modulation of LILR function could be used as a means to modulate TLR activity.

## 1. Toll-Like Receptor Activity and Antigen Presenting Cell Phenotype

**Figure 1:**
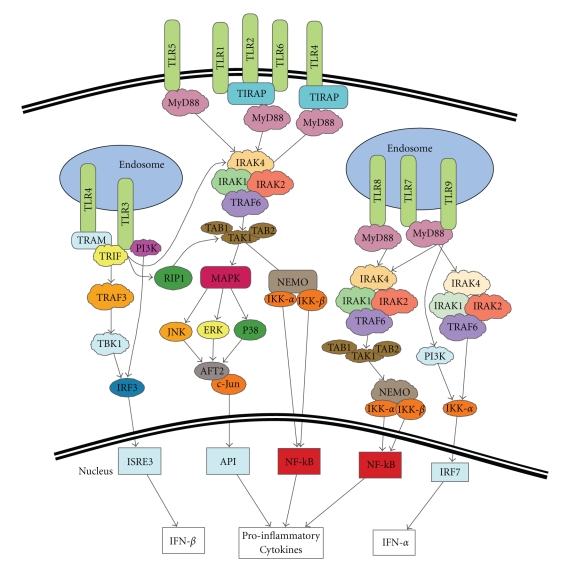
TLR signalling pathways: All TLRs except TLR3 share a common initial signalling pathway through MyD88 to the IRAK1/2/4TRAF6 complex. TLR3 uses TRIF to activate the IRAK1/2/4TRAF6 complex. From here, TLR1–6 are able to signal via MAPK and NEMO to activate API and NF-*κ*B, respectively, and promote transcription of proinflammatory cytokines. TLR8 also follows the same pattern but is only able to signal via NEMO, not MAPK. TLR7/9 are also able to activate NEMO and additionally IKK-*α* to promote IFN-*α* production. In addition to the MAPK and NEMO pathways, TLR3 is also able to signal via TRAF3 to promote IFN-*β* production [25–27].

Toll-like receptors (TLRs) are pattern recognition receptors with the ability to detect microbial products. They can provide the initial danger signal required to alert the body to bacterial or viral infection, playing a pivotal role in the activation of both innate and adaptive immune responses. TLRs recognise pathogen-associated molecular patterns (PAMPs), including lipopolysaccharide (LPS), a membrane component of gram negative bacteria; lipoproteins, functional proteins anchored to the cell membrane; flagellin, a major component of flagellum; heat shock proteins which are highly expressed during cellular stress and microbial nucleic acids [1, 2]. Ligation of individual or complex TLRs can activate different signalling pathways. Most TLRs signal via MyD88 resulting in NF-*κ*B or MAPK activation (Figure 1), ultimately leading to the transcription of genes associated with antimicrobial defence such as inflammatory cytokines, costimulation molecules, MHC, and nitric oxide (NO) [3, 4].

TLRs are widely expressed on immune cells and possess distinctive functions dependent on cell type and signalling pathway [5, 15]. We will focus on their effects on dendritic cells and macrophages, which can act as professional antigen presenting cells (APCs). The expression profile of TLRs on APCs varies between subsets (Table 1), which include plasmacytoid DCs (pDCs), myeloid DCs (mDCs), monocyte-derived DCs (moDCs), and macrophages. TLR signalling in these immune cell subsets triggers an activation programme that includes cytokine secretion; pDCs secrete type I interferons (IFN-*α*) which have a fundamental antiviral function through the recruitment of immune cells and their role in T cell differentiation [16]. In contrast, mDCs and moDCs primarily secrete IL-12, a potent proinflammatory cytokine involved in T cell differentiation and NK cell activation [17]. Macrophages, which provide an initial antimicrobial response, also secrete IL-12, NO, and TNF-*α* which play an important role in apoptotic cell death and bacterial lysis [18]. In addition to the production of soluble cytokines and chemokines, upregulation of various cell surface markers such as the costimulation molecules and MHC required for antigen presentation is also observed following TLR activity [19, 20]. Of these, the best defined costimulation molecules are CD80, CD86, and B7-H1. Both CD80 and CD86 bind to CD28 on T cells to provide an activating signal, whereas B7-H1 binds to PD1 to generate an inhibitory signal [21]. The type of costimulation molecules upregulated and cytokine profile secreted by an APC is thought to be determined by the activation signal given by individual TLRs, and can determine the nature of the downstream immune response [20, 22, 23]. 

Upon migration to the lymph nodes, activated DCs present microbial antigens to prime a specific T cell response. MHC class II-restricted antigens are recognised by T helper (Th) cells, which then secrete proinflammatory cytokines to recruit effector cells and aid in B cell maturation. Th cell responses are defined by their cytokine secretion profile. For example, the cytokines IL-12 and IFN-I dominate Th1 responses, resulting in the recruitment of proinflammatory effector cells and the clearance of infection. Th17 cells are also proinflammatory and through the secretion of IL-17 and IL-22 stimulate, for example, the secretion of antimicrobial proteins from other effector cells. For Th2 responses the cytokines IL-4 and IL-25 dominate, resulting in inhibition of proinflammatory cytokine secretion and further proliferation. Th1 responses are required for pathogen clearance. However, during excessive immune activation, Th1 responses may damage host tissues, raising the possibility of pathology or chronic inflammatory diseases. In order to prevent this, populations of Th2 and regulatory T cells (Tregs) are required, the effects of which regulate the Th1 response [24]. 

T cell polarisation to either Th1, Th2, or Th17 profile is dependent on the type of costimulation molecules and cytokines expressed by the APC [28, 29], which is in turn determined by TLR signalling profile. In this respect, signalling through individual or different combinations of TLR may control the nature of the adaptive immune response towards any given antigen [30]. TLR4, TLR7, and TLR8 are typically thought to trigger definitive Th1 responses, whereas TLR2 has been implicated in inducing Th2 responses. However, recent studies have demonstrated that simultaneous coligation of TLR2 with other TLRs alters the signalling profile and ultimately the DC signature required for T cell polarisation, and may dampen down TLR4 mediated responses [31]. Coligation with accessory molecules such as dectin-1 is also thought to play a part in polarising immune responses [32], and Eisenbarth et al. have demonstrated that levels of antigen stimulation may impact on DC signatures, with high levels of LPS triggering a Th1 response, and low levels a Th2 response [31, 33, 34]. 

**Figure 2:**
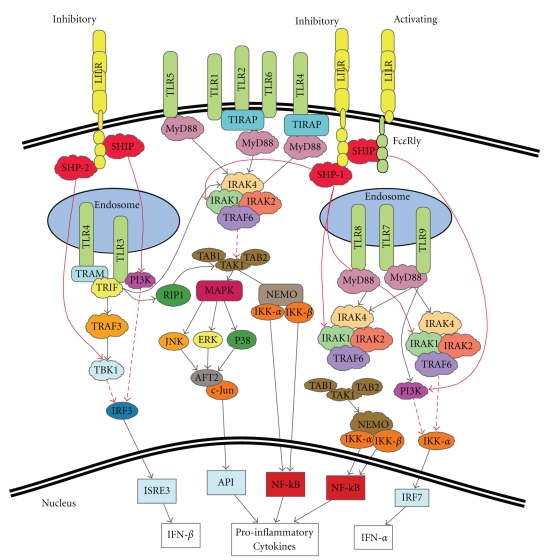
Possible pathways for LILR regulation of Toll-Like Receptor Signalling Pathways: There are several possible mechanisms of inhibition LILR receptors may employ to regulate TLRs. SHP-1 has been shown to associate with IRAK1 and inhibit further downstream signalling. Similarly, SHP-2 and SHIP have been shown to inhibit downstream signalling of TBK-1 and PI3K, respectively, thereby dampening down the production of proinflammatory mediators [35–38].

As an invading pathogen is likely to possess multiple PAMPs and trigger several TLRs, any infection would be predicted to elicit multifaceted T cell responses. Furthermore, as TLRs are known to complex with other receptors and recruit adapter molecules involved in signalling cascades, it is possible that some of these interactions function as regulatory mechanisms. The mechanisms involved in TLR regulation are only just becoming clear. Scavenger receptor A (SRA or CD205) [39], and single Ig IL-1R-related molecule (SIGIRR)/Toll IL-1R8 have been described as candidate regulatory proteins [40]. Members of the leukocyte Ig-like receptor family (LILR, also known as ILTs, LIR, CD85, and MIR [41–43]) have also become a focus for investigation after being shown to exert a powerful inhibitory effect on TLR functions [44] (Figure 2).

## 2. LILR and Their Murine Equivalents (PIR and LILRB4)

LILRs are a family of innate immune receptors that are predominantly expressed on antigen-presenting cells and B cells. The eleven members of the human LILRs family are split into three distinct groups: activating, inhibitory and soluble. The LILR classed as inhibitory (LILRB1-5) have a cytoplasmic tail containing 2–4 immunoreceptor tyrosine-based inhibitory domains (ITIMs). LILRs classed as activating (LILRA1-2, 4–6) lack any signalling motif, but instead possess a charged arginine residue which enables association with the adaptor protein Fc*ε*RI*γ* [45]. To date, this is the only identified adaptor molecule shown to associate with LILR, although it is possible that other adapter molecules are capable of this association. Signalling is then directed through the Fc*ε*RI*γ*-associated immnuoreceptors tyrosine-based activatory domains (ITAMs). Despite this classification, the so-called activating group contains receptors with the ability to exert inhibitory effects, a phenomenon that has been observed for several ITAM-bearing immune receptors and is thought to be related to strength of signalling [46]. 

LILRs are conserved throughout evolution and to date homologues have been identified in rodents [47], chickens [48], and cattle [49]. In rodents, LILR equivalents are known as paired immunoglobulin-like receptors, a family that contains multiple activating receptors (PIR-A) but only one inhibitory receptor, PIR-B [47, 50–52]. PIRs were classified as LILR homologues due to their similarities in genetic sequence and location, expression profile, structure, and function [53–55]. They have since proved to be an effective tool to examine the role of these receptors [51]. A further murine homologue, LILRB4 was previously known as gp49B [56]. 

Similar to TLRs, LILR expression varies between APC subsets (Table 1). MDC and moDCs express LILRA2, LILRB1, LILRB3, and LILRB4, whereas pDCs express LILRA4, LILRB1, and LILRB4 [6, 57]. LILRB1, LILRB4 and LILRA4 expression is decreased upon DC maturation [6, 58]. Individual receptors exert their regulatory function in a variety of ways. Upregulation and/or cross-linking of LILRB2 inhibits the upregulation of co-stimulatory molecules on APCs, resulting in T cell anergy [59]. In contrast, signalling through LILRA2 inhibits the upregulation of CD1b, HLA-DR, CD40, CD80, CD86 and, CD206, and prevents effective T cell activation and proliferation [60]. Unlike LILRB2, which inhibits APC effector functions by downregulating costimulation molecules, LILRB4 has recently been shown to inhibit APC response to bacterial infection by upregulating IL-10 production and subsequently downregulating IL-8 secretion. Ligation of LILRB4 does not appear to affect the expression of costimulation molecules [61]. However, Lu et al., demonstrated that LILRB4 was able to inhibit TNF-*α* release via inhibition of Fc*γ*RI signalling [62], in line with the ability of murine LILRB4 to inhibit inflammatory cytokine production [63]. LILRB4 has also been shown to be highly expressed in patients with malignancy, and this receptor is thought to play a critical role in the induction of tolerance. However, it is not known whether LILRB4 expression on malignant cells is induced by regulatory T cells, or if the LILRB4 expression binds to a T cell ligand rendering them anergic [63].

## 3. LILR Ligand Specificity and Signalling

Members of the LILR and PIR families have been shown to be involved in bacterial engagement [64]. Both PIR-A1 and PIR-B, and their corresponding human homologues LILRB1 and LILRB3 have been shown to bind bacteria including *E. coli, H. pylori, *and *S. aureus* [64]. The bacterial ligand(s) involved in this interaction are yet to be determined and individual receptors vary in their bacterial specificity. However, the most thoroughly characterised ligand for LILR is MHC class I (MHC-I). Unlike other MHC-I-specific receptors, LILRs show a broad specificity for classical and nonclassical forms of MHC-I; LILRB1 and LILRB2 bind all classical MHC-I as well as some nonclassical alleles [65]. LILRB2 has been shown to bind CD1d, which is an MHC-I-like molecule able to present nonprotein antigens to T cells [66]. CD1d is usually recognised by the TCR receptor of NKT cells, which results in the activation of proinflammatory effector cells and target cell lysis. LILR modulation of CD1d activity may therefore be of particular importance in bacterial infections, which can result in an overaccumulation of lipids in the infected cell [66, 67].

Inhibitory LILRs have been shown to exhibit their functions both independently and in association with activating receptors such as TLRs. Inhibitory LILR carry their own signalling motifs, varying from 2 to 4 ITIMs in their cytoplasmic tail. Variation in the number of ITIM domains has been proposed to result in signal amplification or the recruitment of alternative signalling molecules [7]. Upon activation phosphorylated tyrosines within an ITIM become docking sites for either the Src homology 2 domain-containing phosphatase 1 (SHP-1) or SHP-2, or the SH_2_ domain-containing 5′ inositol phosphatise (SHIP) [58]. These phosphatases then dephosphorylate key molecules further downstream in the cascade or those involved in the ITAM signalling of activating receptors, with consequent inhibitory effects [7]. SHP-2 is particularly important in both positive and negative regulation of cellular differentiation [58]. Although yet to be fully defined, these signalling patterns are likely to be found mimicked in the modulation of TLR activation [68, 69].

As mentioned previously, activating LILR has a positively charged arginine residue within the cytoplasmic domain, which enables association with adapter proteins, such as Fc*ε*RI*γ*. When activated, tyrosine molecules in the ITAM domain of Fc*ε*RI*γ* are phosphorylated by protein tyrosine kinases of the Src family kinases, creating binding sites for further signalling molecules, such as zap70 or syc [7]. The recruited signalling molecules may differ depending on cell type, and therefore LILRs may be involved in modulating a wide range of intracellular signalling pathways [70].

### 3.1. LILR-Mediated Control of TLR Functions

LILR-mediated control of TLR activity has been documented for several different bacterial infections. In the case of *S. aureus*, LILR and PIR-B receptors can bind the pathogen in conjunction with TLR2 and trigger the release of inhibitory cytokines such as IL-10 [64]. Inhibition of TLR2 signalling by PIR-B was confirmed in a study of PIR-B^−/−^ mice, where an excessive Th2 response was observed, coupled with impaired DC maturation. This inhibition of DC maturation was thought to arise from the absence of PIR-B regulation of a common signalling pathway used by IL-3, IL-5, and GM-CSF [71]. 

Evidence of a role for LILR (and their murine homologues) providing a counterbalance to TLR activity is most strikingly illustrated by the high mortality rate of *Salmonella* infection for mice lacking the inhibitory receptor PIR-B [72, 73]. Interestingly, instead of the exacerbated immune responses that might have been expected in the absence of an inhibitory receptor, PIR-B-deficient mice were actually more susceptible to *Salmonella* infection, caused by a decrease in phagosomal oxidant production, necessary for bacterial lysis within lysosomes [72]. 

Mycobacterium *leprae* infection can result in tuberculoid (T-lep) or lepromatous leprosy (L-lep). Patients with T-lep typically display a localised form of disease, with effective bacterial clearance. In L-lep, patients suffer from disseminated disease, with large numbers of bacilli. Although the factors that influence disease course are currently unknown, polymorphisms in TLR2/1 are thought to play a role [60]. In a study by Bleharski et al., gene expression analysis identified an up to 5.4-fold overexpression of LILRA2 in skin lesions of L-lep, compared to T-lep [73]. Infected macrophages stimulated with LILRA2 ligands showed a 40% reduction in antimicrobial responses, indicating that LILRA2 also inhibits antimicrobial functions in macrophages [73]. Furthermore, ligation of LILRA2 considerably reduced IL-12 production by TLRs, skewing cytokine activity towards a Th2-biased response. This is important as the immune response in T-lep lesions (where infection is generally contained then cleared) is Th1-biased, whereas L-lep with its higher bacterial loads and disseminated infection is Th2-biased. Therefore, it is possible that the overexpression of LILRA2 in L-lep results in an inadequate Th2-biased immune response and a more severe form of disease [60]. LILRB5, LILRB3, and LILRA3 are also overexpressed in L-lep patient lesions, although their relevance in infection has yet to be determined [70]. 

Signalling through different TLRs has been shown to result in different LILR expression profiles [6, 61, 74]. In human cells the inhibitory receptors LILRB2 and LILRB4 were upregulated following Salmonella infection, an effect which appears to be mediated largely by LPS recognition, as activation of LILRB4 also occurred by both heat-inactivated *Salmonella* and *Salmonella* LPS [61]. In this respect, LILRB4 may play an important role in TLR4 regulation. Similar relationships exist for other LILR and TLR: LILRA4 has been shown to regulate TLR7/9 activity in pDC and LILRA2 has been shown to inhibit TLR4-mediated activity.

In viral infections, the pDC subset plays an important role in mediating antiviral immunity upon activation by pathogenic ligands. TLR7/9, together with TLR3 and TLR8 are localised in endosomal/lysosomal compartments [75], where their activation leads to the production of TNF-*α* and IFN-I, a group of potent antiviral cytokines. The newly-characterised receptor LILRA4 is expressed only on pDCs, where it appears to play an important role in the control of their activity in response to viral TLR stimulation. Following recognition of its ligand, tetherin (also known as BST2), LILRA4 downregulates TLR7 and TLR9-mediated production of IFN-*α* and TNF-*α*, and decreases calcium mobilisation [76]. However, LILRA4 activity does not affect the maturation of the cell, as upregulation of CD80/CD86 is still observed. LILRA4 is also able to inhibit TLR7 and TLR9 signalling after prior antigen stimulation, but is selectively downregulated upon pDC activation [76, 77]. Similarly in murine models, PIR-B has been shown to inhibit TLR9-mediated signalling via Brutons tyrosine kinase (Btk) phosphorylation, which subsequently inhibits NF-*κ*B activation [78].

## 4. Modifying TLR Activity through LILR Signalling

Given the potent effects of LILR on TLR activity, there is the potential in future to use these receptors as a tool for therapeutic modulation of TLR signalling. For example, inhibitory LILR could be triggered by their highest affinity self-ligand, HLA-G [70]. This nonclassical MHC-I allele has a restricted distribution of expression, limited to placental trophoblast cells and thymus epithelial cells, but is overexpressed in certain pathologies including nonrejected allografts, HIV infection, and tumours [79, 80]. HLA-G expression is known to trigger the upregulation of LILR [80]. HLA-G also has a natural tendency to form disulphide-bonded dimers which in turn enhance binding to LILRB1 and LILRB2, resulting in enhanced immunosuppressive effects [81]. Thus, there is therapeutic potential for recombinant HLA-G to be used to downregulate TLR effects through enhancing LILR activity. 

Enhancing LILR expression would be expected to exert a further dampening effect on TLR activity. Expression levels of LILR can also be enhanced by certain agents; Vitamin D3, Dexamethasone, and niflumic acid have been shown to up-regulate the expression of LILRB2 and LILRB4 on DCs, which is seen with an accompanying increase in IL-10 secretion and Treg differentiation. Although the exact mechanisms involved in tolerance induction in these studies are yet to be fully elucidated, high expression of LILRB4 is thought to be strongly associated with inhibition of NF-*κ*B activation [82, 83]. Recently a study examining the effects of 1,25(OH)_2_D_3_ on DCs demonstrated that this agent is able to up-regulate LILRB4 on moDCs and mDCs but not pDCs. This may be due to the fact that the normal expression levels of LILRB4 are markedly higher on pDCs than mDCs [84]. Further to this, the IDO activity in tryptophan (trp) deficient cells was used in a study recently to define mechanisms of DC tolerance and induction of Tregs. Brenk et al. found that upon DC tolerisation, high levels of LILRB3 and LILRB4 were upregulated. However, replacement of trp was unable to reverse the tolerogenic conditions, and DCs continued to stimulate T cells to differentiate into a regulatory phenotype. Furthermore, only by using anti-LILRB4 antibodies were they able to restore any function to the DCs and subsequently the T cells. The authors predict that DC regulation induced in this manner may affect the epigentics of foxP3 gene transcription and provide antigen-specific Treg cells, therefore providing a mechanism open to therapeutic manipulation [85]. 

Given the powerful inhibitory nature of LILR, it may be possible to modulate the expression of these receptors prior to or in conjunction with chemotherapies in order to enhance treatment efficacy. Blocking the inhibitory functions of LILR has been demonstrated recently in a study by Morel and Bellón, in which amoxicillin was shown to have the ability to interfere with LILRB1 recognition of MHC class I on NK cells, a finding which could potentially have a large impact on tumour immunology and therapeutics [86]. With further research, the bacterial interaction with LILR will most likely prove fundamental in defining regulatory pathways involved in TLR pathogen responses. As more ligands are discovered for these inhibitory receptors, the potential for development of novel therapies targeting specific LILR allows the possibility of shaping immune responses in disease settings.

## Figures and Tables

**Table 1:** Expression profile of TLR and LILR on different APC subsets: this table depicts the known expression levels of TLRs and LILR on subsets of APCs. + denotes high expression, −/+ denotes weak expression, and − is no expression. ? is used where expression levels are yet to be determined [5–14].

	Monocytes	Macrophages	pDC's	mDC's	moDC's
TLR1	+	+	−/+	+	−/+
TLR2	+	+	−	+	+
TLR3	−	−	−	+	+
TLR4	+	+	−	+	+
TLR5	+	+	−	−	−
TLR6	−/+	+	−/+	−	−
TLR7	−	+	+	+	+
TLR8	+	+	−	+	+
TLR9	−	−	+	+	+
TLR10	−	−	−/+	−	−
LILRA1	−	+	−	−	−/+
LILRA2	+	+	+	+	+
LILRA3	+	−	−	−	−
LILRA4	−	−	+	−	−
LILRA5	+	−	−	−	−/+
LILRA6	?	?	?	?	?
LILRB1	+	+	+	+	+
LILRB2	+	+	+	+	+
LILRB3	+	+	+	+	+
LILRB4	+	+	+	+	+
LILRB5	−	−/+	?	−	−
